# Goyal-Naqvi Syndrome (Concurrent Trisomy 10p and Terminal 14q Deletion): A Review of the Literature

**DOI:** 10.7759/cureus.16652

**Published:** 2021-07-26

**Authors:** Chanan Goyal, Vivek Goyal, Waqar M Naqvi

**Affiliations:** 1 Physiotherapy, Datta Meghe Institute of Medical Sciences, Wardha, IND; 2 Paediatric Physiotherapy, Government Physiotherapy College, Raipur, IND; 3 Department of Anesthesiology, Shri Balaji Institute of Medical Science, Raipur, IND; 4 Community Physiotherapy, Mahatma Gandhi Mission School of Physiotherapy, Aurangabad, IND; 5 Community Physiotherapy, Mahatma Gandhi Mission Institute of Health Sciences, Navi Mumbai, IND

**Keywords:** goyal naqvi syndrome, goyal-naqvi syndrome, trisomy 10p, terminal 14q deletion, rare genetic disorder, rare syndrome

## Abstract

Goyal-Naqvi syndrome (GNS) is a newly documented clinical entity that comprises trisomy 10p and terminal 14q deletion, though trisomy 10p and terminal 14q deletion have been discovered as distinct conditions in 1974 and 1997, respectively. Nevertheless, to date, the total number of reported cases of each of these conditions is estimated to be in double digits. Both manifest as a constellation of features like craniofacial dysmorphism, hypotonia, intellectual impairment and global developmental delay. Characteristic facies include protruded forehead, hypertelorism, epicanthic folds, down slanting palpebral fissures, flat nasal bridge, long philtrum, thin upper lip, carp-shaped mouth, retro-micrognathia and low set ears. Besides, trisomy 10p is strikingly associated with clinodactyly and camptodactyly which aids in clinical diagnosis, apart from other musculoskeletal deformities like hip dysplasia and pes planus. Intersex conditions have been found to commonly co-exist. As other systems also display involvement frequently, trisomy 10p is a discernible multiple congenital anomalies/mental retardation (MCA/MR) syndrome. On the other hand, with terminal 14q deletion, increased risk of certain types of cancer was predicted as specific tumor suppressor genes are lost in the deletion and thus, screening was recommended. Genetic workup using techniques like fluorescence in situ hybridization (FISH), spectral karyotyping (SKY) and chromosomal microarray-based comparative genomic hybridization (CGH) was found to be helpful in diagnosis of trisomy 10p and 14q deletion. Prenatal diagnosis of these conditions has been well documented too. Intrauterine growth retardation has been observed to be related to trisomy 10p. There is a paucity of literature on the management of children diagnosed with trisomy 10p or with terminal 14q deletion. Although management of a child diagnosed with concomitant occurrence of trisomy 10p and terminal 14q deletion by a multidisciplinary approach emphasizing physiotherapeutic intervention has shown remarkable improvement in motor skills, the care of children diagnosed with these genetic aberrations needs further investigation. Documentation of more such cases will help to expand phenotypic spectrum for early identification and to delineate natural history for a life span approach. Early identification and intervention facilitate tapping of the maximum neuroplastic potential for better neurodevelopmental outcomes. We present a review of current literature on this novel syndrome to identify gaps in knowledge to build future research.

## Introduction and background

Goyal-Naqvi syndrome (GNS) is a recently discovered genetic disorder that is a rare combination of two specific aberrations. GNS refers to a concomitant occurrence of an extra copy of genetic material on the short arm of chromosome 10 (trisomy 10p) and deletion of genetic material in the terminal region on the long arm of chromosome 14 (terminal 14q deletion) [1]. Trisomy 10p can be partial or complete and is rare to be diagnosed. According to a study published a decade ago, only 58 cases of trisomy 10p were documented by the year 2011 [2], though the first description of the same, dates back to 1974 [3]. Even more rare is the occurrence of terminal 14q deletion, as just 20 cases had been reported in the literature since its discovery in 1997 [4], in accordance with a study published in the year 2009 [5].

The clinical features of GNS are a combination of manifestations that characterize trisomy 10p and terminal 14q individually. Striking features include hypotonia, intellectual impairment and developmental delay in all domains, namely motor, language, social and adaptive skills. Craniofacial dysmorphism included atypical skull shape, elongated face, protuberant forehead, widely spaced eyes, down slanting palpebral fissures, broad nasal bridge with bulbous nasal tip, low-set ears, long philtrum, thin upper lip, microstomia and retro-micrognathia. Camptodactyly and atypical palmar creases were the classical features present in the hands. Other musculoskeletal features included widely abducted hips, bilateral hyperextension of knees and pes planus. Besides, the child displayed frequent extraneous movements of the head and neck [1].

Although there is rich literature on phenotype and diagnosis of patients with trisomy 10p and terminal 14q deletion, a dearth of studies on the management of these patients is present. However, the female child reported with GNS was found to benefit from physiotherapy intervention [1]. We document a review of presently available literature on trisomy 10p and terminal 14q deletion that form the components of Goyal-Naqvi syndrome that provides a base of what is known to reveal gaps in knowledge that would help in laying recommendations about research in unexplored avenues. 

## Review

PubMed and ScienceDirect databases were searched with keywords Goyal-Naqvi syndrome, trisomy 10p and terminal 14q deletion. Relevant studies in English were considered for the review.

Trisomy 10p

Prevalence

Trisomy 10 is not as uncommon as trisomy 10p. Trisomy 10p can be either partial or complete. According to a study published in 2012, only five cases of complete trisomy 10p had been documented in the literature till then [6]. The estimated number of total cases reported to date is in double digits as by the year 2011, only 58 cases of trisomy 10p were documented since its first discovery [2].

Phenotype

Trisomy 10p is characterized by craniofacial abnormalities including dolichocephaly/ microcephaly, long face, broad forehead, widely spaced eyes, depressed nasal bridge, high arched palate and prominent ears as well as neurological deficits comprising neurodevelopmental delay and hypotonia [6-23]. Multiple congenital abnormalities are frequently observed namely cleft lip and cleft palate, cardiac manifestations including atrial septal defect, ventricular septal defect, patent ductus arteriosus, musculoskeletal deformities including barrel-shaped chest, genu recurvatum, talipes equinovarus and flexed large joints, integumentary system involvement like marbled skin [6-10,12-16,18,24]. Some striking features in the hand that can aid clinical diagnosis are atypical palmar creases like formation of a triangle, camptodactyly and clinodactyly [7,11,14,18-20,24]. The first case report on GNS also reported similar findings in context of hand [1]. It is interesting to note that around 20% of males presenting with duplication of 10p displayed intersex condition [7]. Inguinal/umbilical hernia and genital abnormalities like hypospadias, cryptorchidism were observed in multiple cases [11,12,25]. Turner syndrome was associated with trisomy 10p as documented in a study [26]. Despite variation observed over the phenotypic spectrum, trisomy 10p demonstrates discernible multiple congenital anomalies/mental retardation (MCA/MR) syndrome [11,27,28]. In conformation to the case report on GNS, absence of cardiopulmonary malformations has been observed in other previous studies on trisomy 10p [1,13-15]. One of the studies reported features resembling facio-auriculo-vertebral spectrum in a child with trisomy 10p [29].

Diagnosis

Trisomy 10p with or without other genetic aberrations has been diagnosed prenatally in numerous reports [9,10,30-33]. Oligohydramnios and intrauterine growth retardation (IUGR) were significant findings in prenatal investigations [6].

Most of the documented cases have trisomy for distal one-third of 10p [34]. Different methods used for diagnosis or determining parental origin in various studies were fluorescence in situ hybridization (FISH), spectral karyotyping (SKY), chromosomal microarray-based comparative genomic hybridization (CGH) and restriction fragment length polymorphism (RFLP) analysis [35,36]. A ratio of about 2:1 is estimated between the frequencies of balanced and karyotypically normal offspring from carriers in trisomy 10p [37]. For most children diagnosed with trisomy 10p, the carrier was the mother [1,3,7,10,17,18,20,23,30,38-40].

Terminal 14q deletion

Prevalence

14q deletion can be a linear (interstitial or terminal) or ring chromosome deletion. The total number of terminal 14q deletion cases reported since the first description in 1997 is estimated to be in the double digits [1,4,5].

Phenotype

Phenotypic delineation of terminal 14q deletion comprises hypotonia, global developmental delay, facial dysmorphism (frontal bossing, epicanthic folds, blepharophimosis, thin upper lip vermillion, wide philtrum, triangular/fish-shaped mouth, micrognathia) as was observed in the child with GNS too [1,41-47]. Musculoskeletal manifestations like joint laxity, scoliosis, arthrogryposis multiplex congenita have also been noted as striking features [48]. An extremely mild form of holoprosencephaly was documented with terminal 14q deletion, which is otherwise common with interstitial deletion [49,50]. Clinical features of interstitial 14q deletion that resemble those of terminal deletion are craniofacial dysmorphism like microcephaly, widely spaced eyes, epicanthal folds, long-smooth philtrum and tooth agenesis, apart from neurological manifestations like hypotonia and delayed psychomotor development. Hydronephrosis was a finding in ultrasound examination of kidney too [51]. This was similar to the case reported with GNS where fetal ultrasound revealed edema in unilateral kidney [1]. Chromosome 14q carries tumor suppressor genes which on deletion may inflate vulnerability to renal cancer. *L2HGDH* is suggested to be one of the tumor suppressor genes that is a target of 14q deletion [52]. Besides, higher risk of meningiomas and non-Hodgkin’s lymphoma has been predicted [53,54]. Thus, regular screening with high suspicion should be undertaken in patients with 14q deletion [55].

Diagnosis

In a study, prenatal diagnosis of partial monosomy 14q with partial monosomy 5p was documented. Fetal ultrasound demonstrated IUGR apart from single umbilical artery, nuchal edema and microcephaly at 23 weeks of gestation. Other methods and techniques used, thereafter, were amniocentesis, whole-genome array comparative genomic hybridization on non-cultured amniocytes, quantitative fluorescent polymerase chain reaction (PCR) analysis on parental blood and non-cultured cord blood. The study concluded that such a combination of genetic variability can be gauged prenatally [56]. Prenatal diagnosis of 14q deletion has been documented in other studies also [48,57]. The mother has been reported to be the carrier of the translocation in children with terminal 14q deletion [1,58].

Therapeutic intervention

Supportive medical management like vitamin D, iron and folic acid supplementation for the child diagnosed with GNS has been documented [1]. The physiotherapy intervention based on the principles of neurodevelopmental treatment in which the child actively participates in functional activities was reported to bring significant changes in the motor skills of the child diagnosed with GNS as was objectively measured by gross motor function measure (GMFM). Transitions like supine to sit and sit to stand were facilitated. Vestibular and proprioceptive inputs were given. Stabilizing pressure input orthosis for the trunk was found to be beneficial in maintaining posture that was otherwise difficult due to truncal hypotonia [1]. Previous studies on other rare syndromes have also shown remarkable benefits of physiotherapy and use of orthoses on motor function [59,60]. Besides, genetic counselling of the parents must be undertaken as preconception advice in the future [1].

## Conclusions

Techniques for prenatal diagnosis of an extensive list of genetic disorders are available. Simpler forms of screening tests that predict risk of a wide range of aberrations are available but still need further research to increase sensitivity and specificity so that relevant tests for diagnosis can be applied for the pregnant women who need it. Nevertheless, genetic disorders like GNS are expected to remain largely undiagnosed until postnatal life or infancy owing to their rare occurrence and lack of resources, especially in developing and underdeveloped nations. Thus, identification in the early days of life by high degree of suspicion for a child with dysmorphic facies and developmental delay becomes needful for early intervention to tap the highest possible potential of neuroplasticity of the young brain. Though definitive diagnosis after detailed genetic work-up paves the way for streamlined management and prediction of prognosis, therapeutic intervention must not be delayed while that is awaited. Besides, the description of phenotypic spectrum in the literature on patients with similar genotype helps to delineate guidelines for clinical diagnosis. Definitive management and natural history of this rare syndrome must be documented in future studies.
